# Prolonged fecal shedding of replication-competent virus, lasting immune activation, and intestinal inflammation in a rhesus macaque after experimental SARS-CoV-2 infection

**DOI:** 10.3389/fcimb.2024.1505720

**Published:** 2024-12-18

**Authors:** Kinga P. Böszörményi, Marieke A. Stammes, Zahra Fagrouch, Fidel Acar, Henk Niphuis, Gwendoline Kiemenyi Kayere, Lisette Meijer, Eric J. Snijder, Lia van der Hoek, Ben Berkhout, Willy M. Bogers, Judith M. A. van den Brand, Ivanela Kondova, Babs E. Verstrepen, Ernst J. Verschoor

**Affiliations:** ^1^ Department of Virology, Biomedical Primate Research Centre (BPRC), Rijswijk, Netherlands; ^2^ Molecular Virology Laboratory, Leiden University Center of Infectious Diseases, Leiden University Medical Center, Leiden, Netherlands; ^3^ Laboratory of Experimental Virology, Department of Medical Microbiology and Infection Prevention, Amsterdam UMC, University of Amsterdam, Amsterdam, Netherlands; ^4^ Division of Pathology, Faculty of Veterinary Medicine, Utrecht University, Utrecht, Netherlands

**Keywords:** SARS-CoV-2, COVID-19, rhesus macaque, virus shedding, PET-CT, intestinal inflammation

## Abstract

Infection of an adult rhesus macaque with SARS-CoV-2 led to viral RNAemia in nose, throat, and lungs. The animal also presented extended fecal shedding of viral genomic and subgenomic messenger RNA and replication-competent virus for more than 3 weeks after infection. Positron emission tomography revealed increased intestinal glucose metabolism which was histologically related to inflammation of the ileum. These findings highlight the potential of the virus to cause gastrointestinal infections in macaques like this is also regularly observed in COVID-19 patients and substantiates the probability of virus transmission via the fecal-oral route. This study further adds the importance of nonhuman primates as a valuable animal model to study SARS-CoV-2 infection in humans.

## 1 Introduction

The disease caused by SARS-CoV-2, COVID-19, was initially regarded as a respiratory syndrome. However, it soon became clear that SARS-CoV-2 can also cause symptoms affecting various organ systems (Zhu et al., 2023) While the respiratory tract serves as the primary site of infection, the virus can also trigger gastrointestinal (GI) complications in a significant proportion of patients (Wu et al., 2020; Durairajan et al., 2023). Viral RNA can be detected in feces of infected individuals for a considerable time, often exceeding the detected RNA levels in the respiratory tract (Zhu et al., 2023).High-level expression of ACE2 receptor molecules in the GI tract could explain its susceptibility to SARS-CoV-2 infection (Ribeiro et al., 2023). Alternatively, SARS-CoV-2 may use ACE2-independent entry of cells via the GRP78 cell-surface protein as proposed for the related MERS-CoV, and for other viruses (Chu et al., 2018; Ibrahim et al., 2020; Han et al., 2023). Despite the detection of viral RNA in rectal swabs, the isolation of live virus from these samples has proven cumbersome and reports are scarce (Wolfel et al., 2020; Cerrada-Romero et al., 2022; Ribeiro et al., 2023). Presently, the involvement of SARS-CoV-2 in the pathology of the GI tract is not fully understood and requires further research, particularly in the light of the potential for fecal-oral transmission of SARS-CoV-2.

Nonhuman primates (NHPs), like macaques, are an important animal model for COVID-19 pathogenesis research. Macaques express receptor proteins that are similar to their human counterparts (Melin et al., 2020; Sirakov et al., 2020), are susceptible to infection, and macaques exhibit COVID-19 symptoms and pathology much alike observed in human patients (Caldera-Crespo et al., 2021; Saturday and van Doremalen, 2023). After infection, SARS-CoV-2 induces mild-to-moderate disease in NHPs (Böszörményi et al., 2021; Stammes et al., 2022; Saturday and van Doremalen, 2023), and viral RNA can be detected shortly after experimental infection in the nasal and tracheal area, and in the lungs. However, in macaques, viral RNA is detected sporadically in feces and viral RNA levels tend to be relatively low compared to those measured in the respiratory tract.

We previously reported inflammation of the lungs and brain of rhesus macaques in the post-acute phase of infection (Böszörményi et al., 2021; Philippens et al., 2022). Here, we describe the case of a rhesus macaque with a SARS-CoV-2 infection that coincided with inflammation of the small intestine, accompanied by a prolonged period of fecal shedding of replication-competent SARS-CoV-2.

## 2 Materials and methods

### 2.1 Ethics statement

The study was carried out at the Biomedical Primate Research Centre (BPRC). The BPRC is accredited by the American Association for Accreditation of Laboratory Animal Care (AAALAC) International and is compliant with the European directive 2010/63/EU as well as the “Standard for Humane Care and Use of Laboratory Animals by Foreign Institutions” (National Institutes of Health, ID A5539-01). The study protocol was reviewed and approved by the “Centrale Commissie Dierproeven” (license no. AVD5020020209404) according to Dutch law, article 10a of the “Wet op de Dierproeven” and BPRC’s Animal Welfare Body.

All animal handlings were performed within the Department of Animal Science (ASD) and in accordance with Dutch law. ASD is regularly inspected by the responsible national authority (Nederlandse Voedsel- en Warenautoriteit, NVWA), and the AWB.

### 2.2 Study design

**Figure 1:**
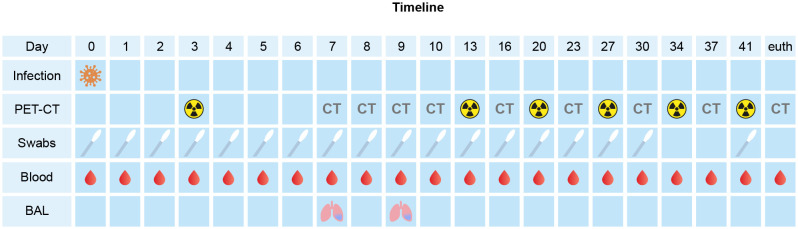
Schematic overview and timeline of the experimental procedures within the study. At the timepoints only CT was performed, it is marked with “CT”. The radioactivity symbol indicates timepoint of PET-CT.

A 10-year-old, healthy, female rhesus macaque (*Macaca mulatta*) with a bodyweight of 6.6 kgs was inoculated with 1x10^5^ TCID_50_ of the SARS-CoV-2/human/NLD/Leiden-0008/2020 strain (GenBank accession number: MT705206.1), which is an early 2020 isolate containing the D614G mutation in the virus’ spike protein. The virus had been propagated twice on Vero E6 cells and titrated on Vero E6 cells in a 96-well format in triplicates. Plates were fixed on day 3 with 10% formalin, stained with crystal violet, and the TCID_50_/mL were determined using the Spearman-Kärber method. On day 0, all animal was exposed to a dose of 1 x 10^5^ TCID_50_ of SARS-CoV-2, diluted in 5 ml phosphate-buffered saline (1x PBS). The virus was inoculated via a combination of the intratracheal route, just below the vocal cords, (4.5 ml) and intranasal route (0.25 ml in each nostril) (Böszörményi et al., 2021). The macaque was monitored for six weeks after infection (**Figure 1**). Body temperature was measured and nasal, tracheal, anal swabs, and blood samples were collected daily for the first 10 days, and subsequently every 3-4 days. Broncho-alveolar lavage (BAL) was performed on days 7 and 9 post-infection (pi.). Hematology and blood biochemistry parameters were measured at days 0, 1, 13 and 42.

During the post-infection follow-up period, computed tomography (CT) data were acquired at multiple timepoints pi. Positron emission tomography (PET)-CTs were performed at days 3, 13, 20, 27, 34 and 41 pi. using [^18^F]FDG as tracer as described previously (Böszörményi et al., 2021; Philippens et al., 2022). For all experimental interventions (intratracheal and intranasal infection, swab collections, blood samplings, and imaging procedures), the animal was fasted overnight and sedated with ketamine (10 mg/kg, ketamine hydrochloride, Ketamin 10%; Alfasan Nederland BV, Woerden, the Netherlands) combined with medetomidine hydrochloride, 0.05 mg/kg (Sedastart; AST Farma B.V., Oudewater, the Netherlands) to induce sedation and muscle relaxation, both applied intramuscularly (IM).

Euthanasia was performed at day 44 pi. by using a method listed in Annex IV of the European Directive 2010/63/EU. The animal was first sedated using 12 mg/kg Ketamin combined with 0.05 mg/kg Medetomidine, followed by intravenous administration of 70 mg/kg pentobarbital (Covetrus, Cuijk, the Netherlands). Then, full necropsy was performed, and tissue samples were preserved for (immuno)histological analysis.

### 2.3 Detection of viral RNA in swabs, blood, and tissues

Nasal, tracheal, anal swab samples were taken at regular time-point, and at the same time, blood samples were collected for PCR analysis. At the end of the study, the animal was euthanized, and tissue samples were collected for viral RNA detection. RNA isolation from swabs and tissue samples was done using QIAamp Viral RNA Mini kit (Qiagen Benelux BV, Venlo, The Netherlands) following the manufacturer’s instructions. Detection of SARS-CoV-2 viral RNA (vRNA) and subgenomic messenger RNA (sgmRNA) was performed following published protocols (Corman et al., 2020; Wolfel et al., 2020). Detection of sgmRNA was used as a proxy of replicating virus (Dagotto et al., 2021). Both assays had a lower limit of quantification of 20 viral RNA copies per reaction and was determined using RNA standard curves that were generated by *in vitro* transcription of the target regions from synthetic DNA.

### 2.4 Virus isolation from fecal samples and NGS analysis

Anal swabs were assayed for the presence of replication-competent virus in Vero-E6 cells (ATCC# CRL-1586). Fecal swabs were put in 1 mL MEM, supplemented with 0.5% bovine serum albumin (BSA), fungizone (2,5 µg/mL), penicillin (100 U/mL), and streptomycin (100µµg/mL) and directly transported to the BSL3 lab, vortexed, and supernatant was clarified by centrifugation at 2800 x *g* for 5 min. Next, 250 µl of the sample was inoculated on Vero E6 cells and incubated at 37˚C in the presence of 5% CO_2_ for 6 days. Cell cultures were screened daily for signs of infection by light microscope and at the same time a sample was taken for PCR analysis. RNA was isolated from the harvested cell culture supernatant and used for NGS analysis using Illumina sequencing as described previously (Ogando et al., 2020).

### 2.5 Imaging

Positron Emission Tomography Computed Tomography (PET-CT) data was acquired, using a MultiScan Large Field of View Extreme Resolution Research Imager (LFER) 150 PET-CT (Mediso Medical Imaging Systems Ltd., Budapest, Hungary). After sedation, the animal was positioned head-first supine (HFS) with the arms up. After the scan, upon return to its home cage, atipamezole hydrochloride (Sedastop, AST Farma B.V., Oudewater, The Netherlands, 5 mg/mL, 0.25 mg/kg) was administrated IM to antagonize medetomidine.

#### 2.5.1 CT

CTs were acquired using a semicircular single FOV scan method, with an exposure of 90 ms and 1:4 binning using 75 kVp, 980 µA CT tube strength. CTs were reconstructed with a voxel size of 500 and 1000 µm. CT image analyses was performed using Vivoquant version 4.5 (InVicro, Boston, USA) (Stammes et al., 2021).

#### 2.5.2 PET-CT

The PET-CTs were acquired under mechanical ventilation in combination with a forced breathing pattern. For anesthetic maintenance, a minimum alveolar concentration of isoflurane (iso-MAC) of around 0,80%-1.00% was used. A 15-minute static PET scan was acquired of brain, thorax and abdomen starting 30 minutes post injection a bolus injection of 102,71 MBq (range 97,21-107,97 MBq) ^18^F-fluorodeoxyglucose ([^18^F]FDG).

Afterwards the emission data was iteratively reconstructed (OSEM3D, 8 iterations and 9 subsets) into a single frame PET image normalized and corrected for attenuation, scatter, and random coincidences using the reference CT and corrected for radioactive decay. The analysis was performed in VivoQuant 4.5 (Invicro, Boston, USA). Based on repeatability parameters for correct interpretation of the results, a standardized uptake value (SUV) was used for robustness (Stammes et al., 2021).

### 2.6 Histopathology and immunohistochemistry

Tissue samples were collected at euthanasia on day 44 pi. (**Supplementary Table 1**) and preserved for histopathology by immersion in 10% neutral-buffered formalin for 72 to 96 h. Specimens for microscopic examination were processed and embedded in paraffin, and sections of 4 µm were stained with hematoxylin and eosin (H&E).

For antigen retrieval, slides were incubated in citrate buffer (pH 6 at 97˚C) for 20 min. Endogenous peroxidase activity was inhibited using Dako Peroxidase Blocking Solution for 5 minutes. Next, after a washing step, the slides were incubated for 15 min with normal goat serum and incubated for 1 hour with the primary antibodies (SARS-CoV/SARS-CoV-2 Nucleoprotein antibody, 40143-T62 Bio-Connect; 1:10 000 diluted). After washing, the slides were incubated for 30 min with the secondary antibody (BrightVision Poly-HRP-Anti Ms-Rb IgG, one component, VWRKDPVO110HRP; undiluted). After a washing step, the chromogen (Vector NovaRed) was added for 8 min to visualize the antigen–antibody binding. Hematoxylin-eosin (H&E) staining was used for general morphology. After immunohistochemical staining, the sections were dehydrated with Alcohol-Xylene and mounted with Pertex^®^ mounting medium (VWR International B.V., Amsterdam, The Netherlands).

## 3 Results

**Figure 2:**
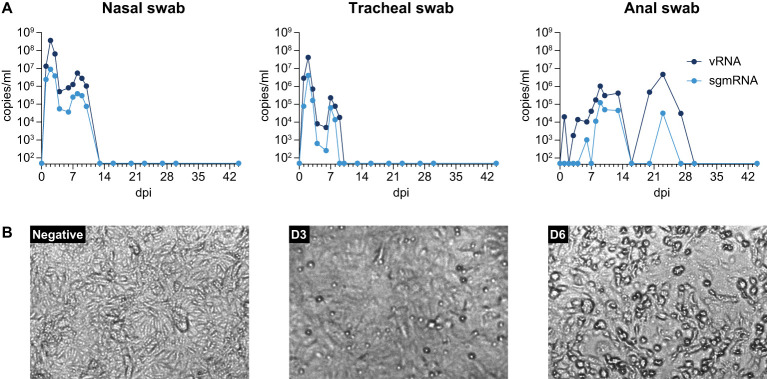
**(A)** SARS-CoV-2 viral RNA and sgmRNA loads in nasal, tracheal, and anal swabs taken throughout the study. **(B)** Virus re-isolation from anal swab collected at day 9 pi. Anal swab was put in 1 mL MEM, supplemented with 0.5% bovine serum albumin (BSA), fungizone (2,5 g/mL), penicillin (100 U/mL), and streptomycin (100µg/mL), vortexed, and supernatant was clarified by centrifugation at 2800 x *g* for 5 min. Next, 250 µl was inoculated onto Vero E6 cells and incubated for 6 days. From left to right: uninfected cells, Vero cells after 3 days and 6 days culturing, respectively.

Following inoculation, no changes in body temperature, hematology and blood biochemistry were measured. SARS-CoV-2 replication was monitored by RT-qPCR (**Figure 2A**). Viral RNA was detected in nose and throat but was undetectable in all blood samples. RNAemia started at day 1 pi. and in both compartments a peak in RNA load was visible around day 2 and a second peak on day 7 and day 8 in the throat and nose, respectively. Then, RNA levels rapidly dropped below the limit of detection within 10 days pi. in the throat and 13 days pi. in the nose. BAL samples were positive at both collection timepoints on days 7 and 9 pi. (7,54 x 10^4^ copies/mL and 2,27 x 10^4^ copies/mL, respectively). Interestingly, viral RNA was also detected in anal swabs from day 1 pi. and remained consistently present until day 27 pi. This pattern contrasts with the vRNA detection in nose and throat samples which fell below the detection limit on day 13 (nose) and day 10 (throat), resembling the temporal dynamics observed in human patients (Zhu et al., 2023). Detection of subgenomic messenger RNA was used as a qualified proxy for monitoring virus replication (Dagotto et al., 2021). SgmRNA was identified in nasal and tracheal swabs from day 1 and remained detectable until days 8-10 pi., while showing the same bi-phasic pattern as the vRNA loads. Additionally, sgmRNA was present in the BAL sample collected on day 7 pi. (3,74 x 10^4^ copies/mL), but not in the BAL fluid collected at day 9 pi., indicating that virus replication in the upper and lower respiratory tract of the macaque started early after infection and continued for a limited time frame.

In anal swabs, vRNA was detected over a period of 30 days, starting at day 1 pi., and detection of sgmRNA started at day 6 pi. and lasted until day 23 pi. This observation suggests that SARS-CoV-2 replicated in the gastro-intestinal (GI) tract for a substantial period. Furthermore, virus replication was substantiated by the successful recovery of infectious SARS-CoV-2 from the anal swab sample collected at day 9 pi. (**Figure 2B**). This was the timepoint with the highest number of sgmRNA copies detected (1.24 x 10^5^ copies/mL). Virus isolation from sgmRNA-positive anal swab samples collected at other time-points was unsuccessful.

**Figure 3:**
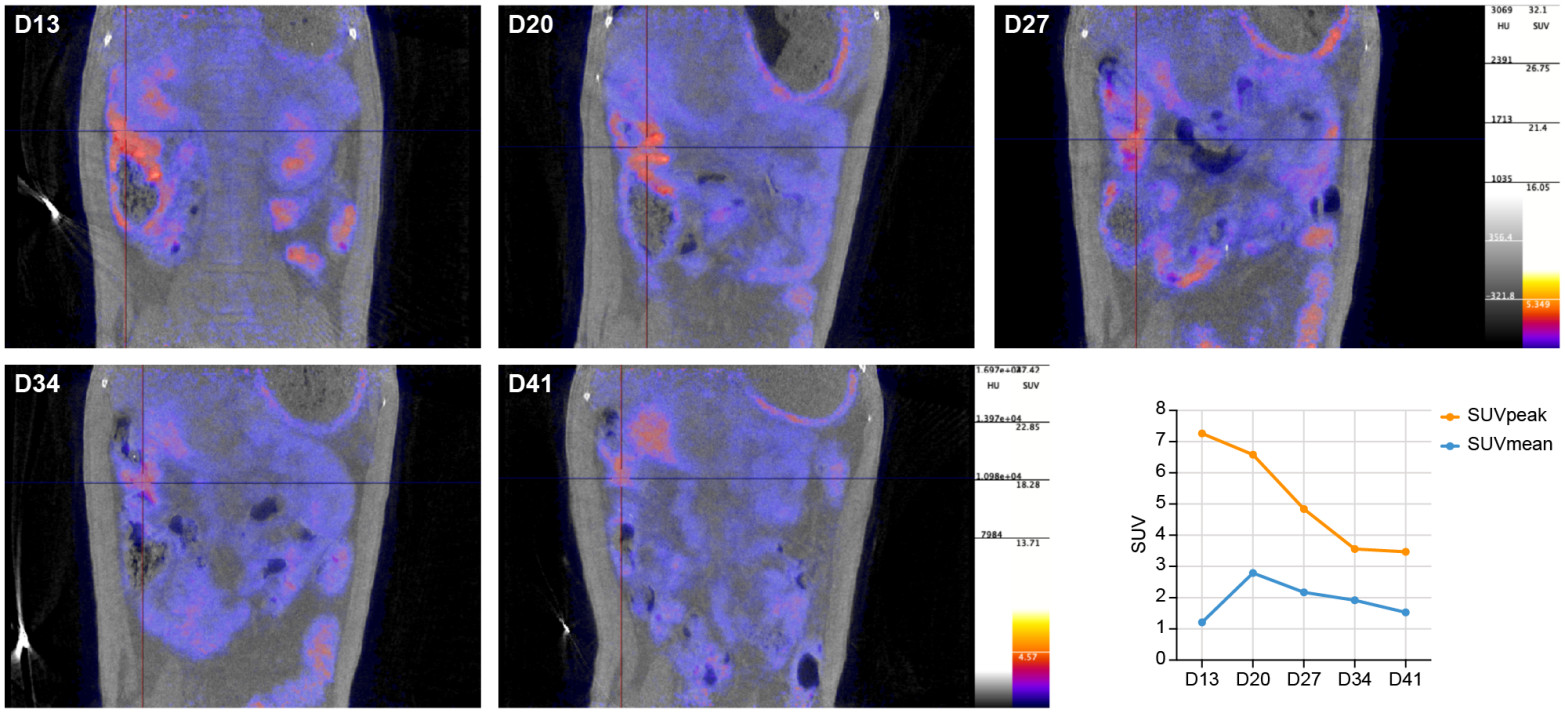
Longitudinal development of metabolic activity by increased [^18^F]FDG uptake in the ileum from day 13 to day 41 post infection. The ileum is marked by the crosshair. The graph (bottom, right) shows the calculated SUVs throughout the study.

Initially, (PET-)CTs were focused on the upper part of the body, excluding the abdominal region. Here, CT revealed mild pathological changes of the lower respiratory tract with abnormalities in the lungs, predominantly ground glass opacities, and an increased uptake of [^18^F]FDG in the mediastinal lymph nodes was detected on the longitudinal chest PET-CTs of the macaque (data not shown). However, the recurring detection of sgmRNA in anal swabs led us to expand our PET-CT investigations to the abdomen, starting on day 13 pi. An increased uptake of [^18^F]FDG was observed, beside the mediastinal lymph nodes, in the ileal part of intestinal tract. As no pre-infection scan was performed, this increase was based on comparison with scans obtained from other healthy animals. Although [^18^F]FDG is not specific for SARS-CoV-2 and is used more for detecting general metabolic activation, the data, along with the long-term detection of sgmRNA in anal swabs, strongly suggest SARS-CoV-2 infection in the intestinal tissues and associated immune activation. In **Figure 3**, the longitudinal development of the metabolic activity of the ileum is presented. The [^18^F]FDG tracer uptake increased from day 13 to 27 pi., and afterwards intensity decreased but remained detectable towards the end of the study. When analyzing the data, this observation was confirmed by the standard uptake values (SUVs). The SUVpeak (max value in 1 mm^3^ spherical volume) reached its highest level on day 13 pi (SUVpeak 7.26), although this peak was observed in only a small portion of the sample. The average value in the region of interest (SUVmean), however, continued to increase until day 20 pi. (SUVmean was 1.21 at day 13 compared to 2.79 at day 20), after which it began to decrease but remained above the initially detected levels (SUVmean of 1.53 at day 41).

SARS-CoV-2 re-isolation was only successful from the anal swab sample collected at day 9. The isolated virus was subjected to NGS analysis, and its genome was compared to the original viral inoculum. Sequence analysis did not reveal specific mutations that could be linked to the enhanced or prolonged virus replication seen in the GI tract of the animal. Two non-silent mutations were identified in the spike (S) protein gene, encoding S151R and D215V substitutions, but each occurred with low frequencies of 8% and 11%, respectively. The importance of these mutations remains currently unclear.

**Figure 4:**
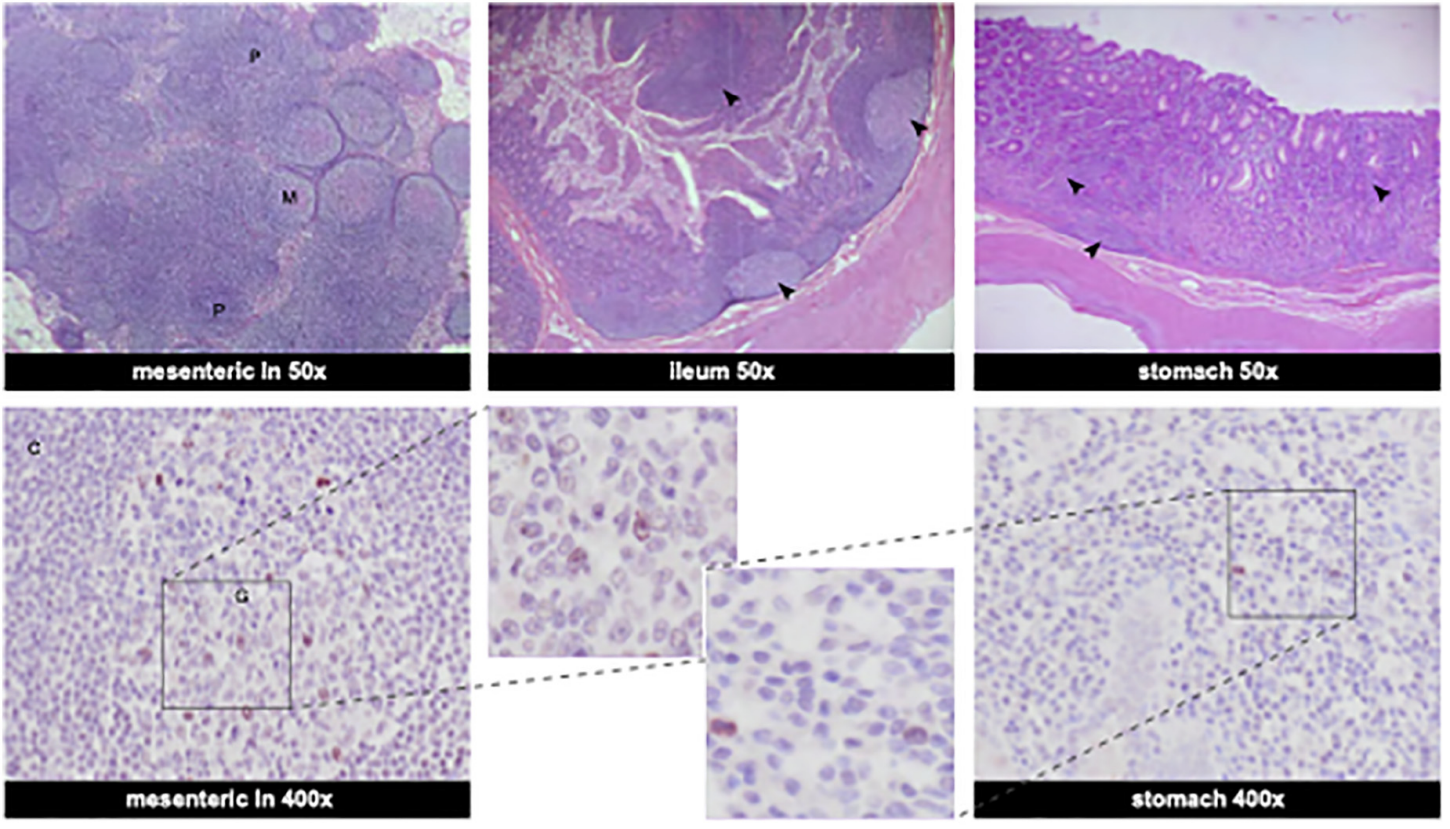
Histological findings in the gastrointestinal organs and viral antigen determined by immunohistochemistry. Top row shows HE stainings at 50x magnification. Bottom row shows SARS-CoV-2 nucleocapsid IHC staining. *Mesenteric lymph nodes:* Numerous large lymphoid follicles are present in the paracortex (P) and medulla (M) of the mesenteric lymph node (HE stainings, 50x). Few cells are positive in the germinal center (G) of the mesenteric lymph node with clear staining of the nucleus (C: cortex) (IHC staining SARS-CoV-2 Nucleocapsid protein, 400x). *Ileum*: The ileum exhibits moderate multifocal lymphoplasmacytic infiltrates in the mucosa and the Peyer’s patches are associated with defined lymphoid follicles with large germinal centers and abundant lymphoid hyperplasia (marked with arrows) (ileum, HE stainings, 50x). *Stomach:* Gastric mucosa is multifocally infiltrated by numerous lymphoplasmacytic infiltrates (marked with arrows) (stomach, HE staining, 50x). Occasional large mononuclear cells are positive in the gastric mucosa (stomach, IHC, 400x). Bottom row, middle: enlarged sections of IHC staining of SARS-CoV-2 nucleocapsid-positive cells from mesenteric lymph nodes and stomach.

Several tissue samples, collected at euthanasia on day 44 pi. (**Supplementary Table 1**), tested positive for vRNA in two independent PCR assays: mesenteric LN (2/2; 1.7 x 10^5^ and 2.4 x 10^5^ copies/gram), bronchial LN (2/2; 1.1 x 10^4^ and 1.3 x 10^4^ copies/gram), and the ileum (2/2; 1.5 x 10^4^ and 1.2 x 10^4^ copies/gram). Despite being positive for vRNA, these tissues tested negative in the sgmRNA assay suggesting a resolved, but widely disseminated SARS-CoV-2 infection. This was confirmed by histological analysis. Histology revealed marked lymphoid follicular hyperplasia of the mesenteric lymph nodes, severe multifocal lymphoplasmacytic gastritis, and moderate lymphoplasmacytic enteritis with marked lymphoid hyperplasia in the ileum. Immunohistochemistry was performed on the same set of tissue samples (**Figure 4**). Viral antigen was visualized in the mesenteric lymph node, with few numbers of cells staining positive for the viral nucleocapsid (N) protein. Similarly, occasional positive stained cells were seen in the stomach. However, in the ileal tissue sample no convincing staining of cells was observed. Thus, IHC did not provide definitive evidence of an actual infection of the ileum, but it cannot be excluded that the viral load in this tissue sample was below the detection threshold of the assay by the time the animal was sacrificed.

## 4 Discussion

Although nonhuman primates are widely used in SARS-CoV-2 vaccine research since the start of the COVID-19 pandemic, our knowledge of the pathology induced by SARS-CoV-2 in NHPs is relatively limited. This precludes a thorough comparison with the observed pathological consequences in human patients and therefore hinders a full appreciation of NHPs as a useful and unique animal model for COVID-19 research.

For that reason, while employing NHPs for vaccine evaluation studies, we also investigated striking clinical and pathological manifestations that were encountered in individual macaques after infection with SARS-CoV-2. We previously reported ongoing virus replication and pathogenesis in NHPs and described inflammation and aggregation of α-synuclein in brain of infected macaques (Böszörményi et al., 2021; Philippens et al., 2022).

In this report we describe prolonged SARS-CoV-2 replication in the GI tract of a rhesus macaque and the isolation of replication-competent virus from an anal swab. These findings were complemented by an active inflammation of the ileum, as was visualized by [^18^F]FDG PET-CT. Notably, this persisted till the end of the study, well after RNA shedding in feces had become undetectable. Inflammation of the ileum was further validated by histological examination, but, despite the presence of viral RNA, evidence for viral antigen in the ileum could not be found by immunohistochemistry.

The detection of vRNA in mesenteric and bronchial lymph nodes, suggestive of a resolved virus infection, was paralleled by the detection of morphological abnormalities and provided further evidence of the wide-spread distribution of the virus (Böszörményi et al., 2021; Philippens et al., 2022).

Finding of SARS-CoV-2 RNA in fecal samples from NHP appears less common than in human patients. Viral RNA is found in the GI tract of up to 59% of COVID-19 patients, but such high numbers are seldomly reported in NHPs (Ng and Tilg, 2020; Guo et al., 2021). A single study describes vRNA detection in feces of 4/4 macaques after SARS-CoV-2 and the re-solation of virus from a fecal sample of one animal, but this study used an intragastric infection route (Jiao et al., 2021). Others, using the intratracheal/intranasal infection route, like in this report, described a single individual macaque with vRNA and sgmRNA detectable in feces but without virus culture (Beddingfield et al., 2022). NHP may not exhibit GI disease symptoms caused by SARS-CoV-2 as frequently as observed in human patients, but these figures may be influenced by the overrepresentation of publications focusing on (hospitalized) patients with severe COVID-19. The detection of replication-competent virus or sgmRNA was only successful in critically ill patients, like immunocompromised or immunosuppressed people and in pediatric patients with an immature immune system (Li et al., 2022; Mamishi et al., 2023).

This case report, described in the rhesus macaque model for COVID-19, draws attention to the possible role of silent human carriers in the spread of SARS-CoV-2 during the COVID-19 pandemic. Here, without displaying clinically apparent COVID-19-related respiratory or GI symptoms, the animal actively shed infectious virus in feces which strengthens the concerns about fecal-oral transmission of SARS-CoV-2 in the human population (Xiao et al., 2020; Boyton and Altmann, 2021).

This report adds further value to the use of animal models in studying SARS-CoV-2 infection. The macaque, a recognized NHP model for numerous human viral diseases, provides valuable insights into the biology of SARS-CoV-2 and its potential to cause long-term pathogenic sequelae in different organs, after SARS-CoV-2 infection has been resolved.

## Data Availability

The raw data supporting the conclusions of this article will be made available by the authors, without undue reservation.
