# Supplementary material for: Prolonged fecal shedding of replication-competent virus, lasting immune activation, and intestinal inflammation in a rhesus macaque after experimental SARS-CoV-2 infection
Source: Front Cell Infect Microbiol. 2024 Dec 18;14:1505720. doi: 10.3389/fcimb.2024.1505720 (PMC11688216; doi:10.3389/fcimb.2024.1505720)
Supplement: Supplementary file 1 [file Table1.docx]

**Supplementary table 1. Tissue samples collected at euthanasia (day 44 pi.).**

|  | Formalin | PCR | -80˚C |
| --- | --- | --- | --- |
| Axillary lymph node | x | x | x |
| Mandibular lymph node | x | x | x |
| Salivary gland | x | x | x |
| Conjunctiva | x |  | x |
| Nasal mucosa | x | x | x |
| Oro/nasopharynx | x | x | x |
| Tonsil | x | x | x |
| Trachea | x | x | x |
| Lung | x |  |  |
| Bronchial LN | x | x | x |
| Mesenteric LN | x | x | x |
| Stomach | x |  |  |
| Spleen | x | x | x |
| Duodenum | x | x | x |
| Jejunum | x | x | x |
| Ileum | x | x | x |
| Cecum | x | x | x |
| Colon | x | x | x |
| Heart | x | x | x |
| Kidney | x | x | x |
| Liver | x | x | x |
| Parotic gland | x | x | x |
| Bone marrow | x | x | x |
